# Changing Patterns of Recent Substance Use in Emerging Adulthood and Their Associations With Cognitive Functions and Mental Health

**DOI:** 10.1016/j.bpsgos.2026.100795

**Published:** 2026-07-24

**Authors:** Lukas Eggenberger, Clarissa Janousch, Lydia Johnson-Ferguson, Markus R. Baumgartner, Tina M. Binz, Denis Ribeaud, Manuel Eisner, Lilly Shanahan, Boris B. Quednow

**Affiliations:** a Experimental Pharmacopsychology and Psychological Addiction Research, Department of Adult Psychiatry and Psychotherapy, University Hospital of Psychiatry Zurich, University of Zurich, Zurich, Switzerland; b Jacobs Center for Productive Youth Development, University of Zurich, Zurich, Switzerland; c Digital Society Initiative, University of Zurich, Zurich, Switzerland; d Department of Global Public Health, Karolinska Institutet, Stockholm, Sweden; e Suchtfachstelle Zurich, Zurich, Switzerland; f Center for Forensic Hair Analytics, Zurich Institute of Legal Medicine, University of Zurich, Zurich, Switzerland; g Institute of Criminology, University of Cambridge, Cambridge, United Kingdom; h Department of Psychology, University of Zurich, Zurich, Switzerland; i Neuroscience Center Zurich, University of Zurich and Swiss Federal Institute of Technology, Zurich, Switzerland

**Keywords:** Addiction, Ecstasy, Hair toxicology, Neuropsychology, Recreational use, Substance use disorder

## Abstract

**Background:**

Emerging adulthood is a developmental period marked by heightened risk for illicit substance use, potentially influencing neurocognitive development and mental health. However, longitudinal and objective data on changing patterns of substance use and their associated neuropsychiatric outcomes remain scarce. Longitudinal monitoring of substance use could inform targeted prevention strategies during this sensitive developmental period.

**Methods:**

This Swiss longitudinal cohort study included 731 emerging adults. Hair concentrations of >100 psychoactive substances and metabolites were assessed at ages 20 and 24 years. Latent profile analysis was used to derive profiles of change in recent substance use. Regression models examined associations with cognitive functioning (attention, working memory, declarative memory, composite score) measured at age 24 using the Cambridge Neuropsychological Test Automated Battery and mental health symptoms assessed at both ages 20 and 24 using the Social Behavior Questionnaire.

**Results:**

Among participants with substance use at either time point (*n* = 440), 5 distinct change profiles were identified: low/decreasers (*n* = 288, 65.5%), cannabis increasers (*n* = 48, 10.9%), club drug increasers (*n* = 40, 9.1%), medical stimulant increasers (*n* = 43, 9.8%), and medical sedative increasers (*n* = 21, 4.8%). Compared with participants with no hair-tested substance use (*n* = 291), cannabis increasers showed lower attention (*d* = 0.34) and elevated anxiety-depressive (*d* = 0.53) and attention-deficit/hyperactivity disorder (ADHD) (*d* = 0.38) symptoms. Club drug increasers performed worse on attention (*d* = 0.41) and composite cognitive scores (*d* = 0.39). Medical sedative increasers showed lower attention (*d* = 0.74), working memory (*d* = 0.58), and composite cognitive performance (*d* = 0.76). Medical stimulant increasers showed markedly elevated ADHD symptoms (*d* = 0.82).

**Conclusions:**

Emerging adults’ profiles of change in recent substance use were associated with specific cognitive and mental health risks.

## Plain Language Summary

During the transition from adolescence to adulthood, people differ in how their substance use changes over time. Using hair samples collected at ages 20 and 24 from >700 young Swiss adults, this study identified 5 distinct patterns of substance use change. Groups showing increasing use of cannabis, club drugs, medical stimulants, or medical sedatives each showed unique differences in cognitive performance levels and mental health symptom changes. These findings highlight that different substances carry different risks and that longitudinal monitoring of substance use during this sensitive period could inform targeted prevention efforts.

Emerging adulthood, spanning ages 18 to 25 years, is a distinct developmental period characterized by major life transitions and evolving social roles (1). From a neurodevelopmental perspective, this period involves continued maturation of the prefrontal cortex, which is responsible for executive control and impulse regulation (2,3). In contrast, subcortical reward-related regions develop more rapidly, creating an imbalance that heightens risk taking and susceptibility to external influences during this developmental period (4,5). The developmental and social changes of emerging adulthood create a context in which experimentation with psychoactive substances and changes in substance use patterns are likely (6,7). The use of psychoactive substances usually peaks during this time (8,9), with potential long-term consequences for cognitive functioning, mental health, and daily function.

### Substance Use Profiles in Emerging Adults

Substance use during emerging adulthood is characterized by dynamic changes and substantial interindividual variability. Although use of alcohol and nicotine products are highly prevalent during this age period (8,10), the current focus is restricted to illegal and nonmedical use of psychoactive substances to isolate developmental trajectories that diverge from socially sanctioned experimentation during this period. Within this category of substances, cannabis use often increases during the transition to early adulthood (11,12). However, individuals differ not only in the intensity of use (13) but also in their trajectory: Some show increasing use, whereas others exhibit decreasing use during this period (14,15). Cross-sectional studies have consistently identified subgroups of young adults engaging in polysubstance use (16, 17, 18), although patterns of use vary considerably over time (13). Despite growing evidence that trajectories differ by substance type, research on distinct longitudinal trajectories for specific psychoactive substances remains limited. For example, a large longitudinal study found that the age at which nonmedical prescription drug use peaks varies by drug class, suggesting distinct developmental profiles for medical stimulants compared with medical opioids and sedatives (19).

Most existing studies either focus on a single substance or rely on retrospective self-reports to assess substance use. Given that polysubstance use is common among young adults (18,20) and that self-reported substance use is prone to recall bias, underreporting, and social desirability effects (21,22), objective, longitudinal assessments that capture multiple substances are needed.

### Why Objective Measurement Matters

Hair analysis offers a robust complement to self-reports by providing an objective, cumulative measure of substance exposure over extended periods, thereby capturing long-term substance use patterns with greater accuracy (23,24). Assessing substance use over extended periods is particularly relevant when considering its potential impact on brain development, as prolonged and cumulative exposure may contribute to structural and functional changes (25, 26, 27). When combined with a polysubstance use approach, hair analysis offers an ecologically valid and accurate assessment of real-world substance use behaviors. This is essential for examining how exposure during a sensitive developmental period may shape cognitive and mental health outcomes.

### Cognitive and Mental Health Consequences of Substance Use

The cognitive and mental health consequences of substance use are not uniform; rather, they vary considerably depending on the specific substance and the frequency, duration, and individual trajectory of use. For example, chronic cannabis use has been associated with reduced attention, memory, and executive functions (28,29), whereas chronic cocaine use has been linked to reduced attention, working memory, and declarative memory performance (30). By contrast, chronic use of 3,4-methylenedioxymethamphetamine (MDMA) (ecstasy) has been associated with lower declarative memory functioning (31, 32, 33). Frequent nonmedical use of opioids (34) and ketamine (35,36) has been linked to reduced attention and declarative memory performance. Long-term misuse of the cough suppressant dextromethorphan (DXM) may involve additional neuropsychological differences, although systematic evidence remains limited (37). Overall, different substances appear to implicate distinct cognitive profiles rather than a single generalized pattern of impairment (38).

This substance-specific heterogeneity extends to mental health outcomes. Cannabis use has consistently been linked to internalizing symptoms, such as anxiety and depression (39,40); externalizing symptoms, such as aggression and attention-deficit/hyperactivity disorder (ADHD) symptoms (41); and psychotic symptoms (42,43). Notably, cannabis products containing high levels of Δ^9^-tetrahydrocannabinol (THC) have been consistently linked to strongly elevated psychosis risk (44). The use of illegal stimulants such as cocaine and (meth)amphetamine is associated with internalizing and externalizing symptoms, including increased affective dysregulation and impulsivity (45, 46, 47, 48), and elevated psychosis risk (49). In contrast, although individuals with MDMA use consistently show internalizing symptoms several days after intake (50), little evidence exists for sustained affective or other psychiatric symptoms (51), with the exception of externalizing behavior such as elevated impulsivity (52). Finally, ketamine use is mainly associated with internalizing and psychotic symptoms (53), and nonmedical use of opioids is predominantly associated with internalizing symptoms (54).

However, much of the existing literature is limited by small clinical samples of individuals with substance use disorders, cross-sectional designs, and reliance on self-reported measures. High-risk clinical samples are also likely affected by selection bias and do not represent the broader population of individuals with substance use, in which recreational use patterns predominate. Critically, few studies have considered changes across multiple substances in a more representative community-based longitudinal framework, limiting the ability to capture naturalistic substance use profiles and how their cognitive and mental health consequences unfold over time.

### Additional Factors Associated With Substance Use, Cognition, and Mental Health

Substance use and its effects on cognition and mental health do not occur in isolation; they are influenced by sociodemographic and lifestyle factors. Previous studies sometimes adjusted for a limited set of covariates, such as age, sex, and education, but did not account for others that may shape substance use patterns and their outcomes. For example, socioeconomic status and migration background are well-established predictors of cognitive performance (55, 56, 57) and mental health outcomes (58,59). Additionally, smoking and frequent alcohol use have consistently been linked to worse cognitive functioning (60,61) and mental health risks (62,63). Prior research also suggests that experience with action video games may enhance test performance on computerized neuropsychological tests (64), which is particularly relevant in younger populations. Accounting for these factors is essential to improve validity and ensure that findings translate meaningfully to real-world settings.

### Aims of the Current Study

To address gaps in prior research, we investigated profiles of change in recent substance use in a large community-based sample of emerging adults, using objective hair toxicology testing conducted at ages 20 and 24. We used latent profile analysis (LPA) to identify the change profiles and assess their associations with cognitive and mental health outcomes. We considered a comprehensive set of covariates, including sex, socioeconomic status, migration background, educational attainment, gaming experience, and concurrent tobacco and alcohol use to enhance the robustness and generalizability of the findings.

## Methods and Materials

### Study Overview and Design

The current study used data from z-proso (the Zurich Project on Social Development From Childhood to Adulthood), a large, longitudinal, community-based cohort study. The initial target sample consisted of a community-representative sample of 1675 children, recruited via random cluster-stratified selection from 90 primary schools in the canton of Zurich, Switzerland (65,66). For the current analyses, we used data on substance use and mental health symptom changes from wave 8 (conducted in 2018; *n* = 1180, mean age ± SD = 20.6 ± 0.4 years) and wave 9 (conducted in 2022; *n* = 1160, age = 24.5 ± 0.4 years). Cognitive performance was measured at wave 9 only.

We assert that all procedures contributing to this work comply with the ethical standards of the relevant national and institutional committees on human experimentation and with the Helsinki Declaration of 1975, as revised in 2013 (67). This study was approved by the Cantonal Ethics Committee Zurich (BASEC #2017-02021) and the Ethics Committee of the Faculty of Arts and Social Sciences of the University of Zurich (Approval Nos. 2018.2.12 [phase 5] and 21.12.13 [phase 6]). All participants provided written informed consent prior to participation and received monetary compensation (Table S1). Reporting in this study follows the Strengthening the Reporting of Observational Studies in Epidemiology (STROBE) guidelines.

### Measures

#### Cognitive Performance

Cognitive performance at age 24 was assessed using the Cambridge Neuropsychological Test Automated Battery (CANTAB) (68), a nonverbal computerized cognitive test battery. Three tasks were included: Rapid Visual Processing (A-prime) for sustained attention, Spatial Working Memory (total errors) for visuospatial working memory, and Paired Associates Learning (adjusted total errors) for visuospatial declarative memory. Working and declarative memory scores were inverted so that higher values indicated better performance. A composite cognitive score was created by summing the *z*-standardized task scores. Participants whose performance indicated insufficient effort (e.g., rapid or random responding) were excluded from analysis (see Figure S1).

#### Mental Health Symptoms

Mental health symptoms were measured at ages 20 and 24 using subscales from the Social Behavior Questionnaire (SBQ) (69). Anxiety-depressive symptoms (e.g., “couldn’t enjoy anything”) and psychotic-like symptoms (e.g., “felt as if the thoughts in my head were not my own”) were self-reported over the past month; ADHD symptoms (e.g., “was easily distracted”) were assessed over the past year. Participants rated symptom frequency on a 5-point scale, ranging from 1 = never to 5 = very often. Change scores were calculated by adjusting the absolute difference for baseline (i.e., age 20) symptoms. The SBQ’s psychometric properties for mental health in the z-proso sample have been established previously (70). Reliability and stability coefficients for this study are detailed in Table S2.

#### Toxicological Hair Testing

Concentrations of substances and their metabolites were measured in hair samples at ages 20 and 24 using liquid chromatography–tandem mass spectrometry. Technical specifications and detection thresholds (limit of detection/limit of quantification [LOQ]) are detailed elsewhere (71,72). Given average hair growth rates, the 3 cm of hair analyzed in this study reflects a detection window of approximately 3 months prior to each study visit, capturing recent exposure rather than continuous use over the full 4-year intervisit period. Notably, THC detection in hair is indicative of regular use (73). For analysis, substances and their metabolites were grouped into categories, and medical opioids were converted to morphine-equivalent concentrations consistent with previous work (34,74) (Table S3). Changes in recent substance use were computed by adjusting the difference between ages 20 and 24 for baseline (i.e., age 20) concentrations. To ensure statistical power, only substance categories with at least 30 individual positive hair test results (above LOQ) at either time point were included. All concentrations were log transformed using log(x + 1) to facilitate linear modeling.

#### Covariates: Sociodemographic and Lifestyle Factors

Participants reported on key demographic variables including sex (0 = male, 1 = female), socioeconomic status (measured using the International Socio-Economic Index of Occupational Status, ranging from 16 = unskilled worker to 90 = judge) (75), educational attainment (1 = below apprenticeship, 2 = apprenticeship, 3 = vocational tertiary, 4 = academic tertiary), and migration background (0 = at least 1 parent born in Switzerland and 1 = both parents born outside Switzerland). Participants also reported on lifestyle behaviors, including their engagement in action-packed violent video games over the past year (1 = never to 7 = daily) and tobacco and alcohol consumption (dichotomized into 0 = never or less than daily and 1 = daily). A more detailed overview of these assessments is presented in Table S4.

### Statistical Analysis

LPA was conducted using substance concentration changes as indicator variables to identify distinct profiles of change in recent substance use. Associations of change profiles with cognitive performance at age 24 (CANTAB) and with changes in mental health symptoms between ages 20 and 24 (SBQ) were analyzed using robust SMDM regression estimates (S-estimator followed by M-estimator, Design-adaptive scale estimation, and a final M-estimator step) (76). Models were first run without covariates and then adjusted for potential confounders, including sex, household socioeconomic status, migration background, highest completed education, experience playing action-packed video games, and daily tobacco and alcohol use (see Table S4 for details). Statistical significance was set at an alpha level of 5%, and all analyses were performed using R version 4.3.2 (77).

## Results

### Participants

**Figure 1:**
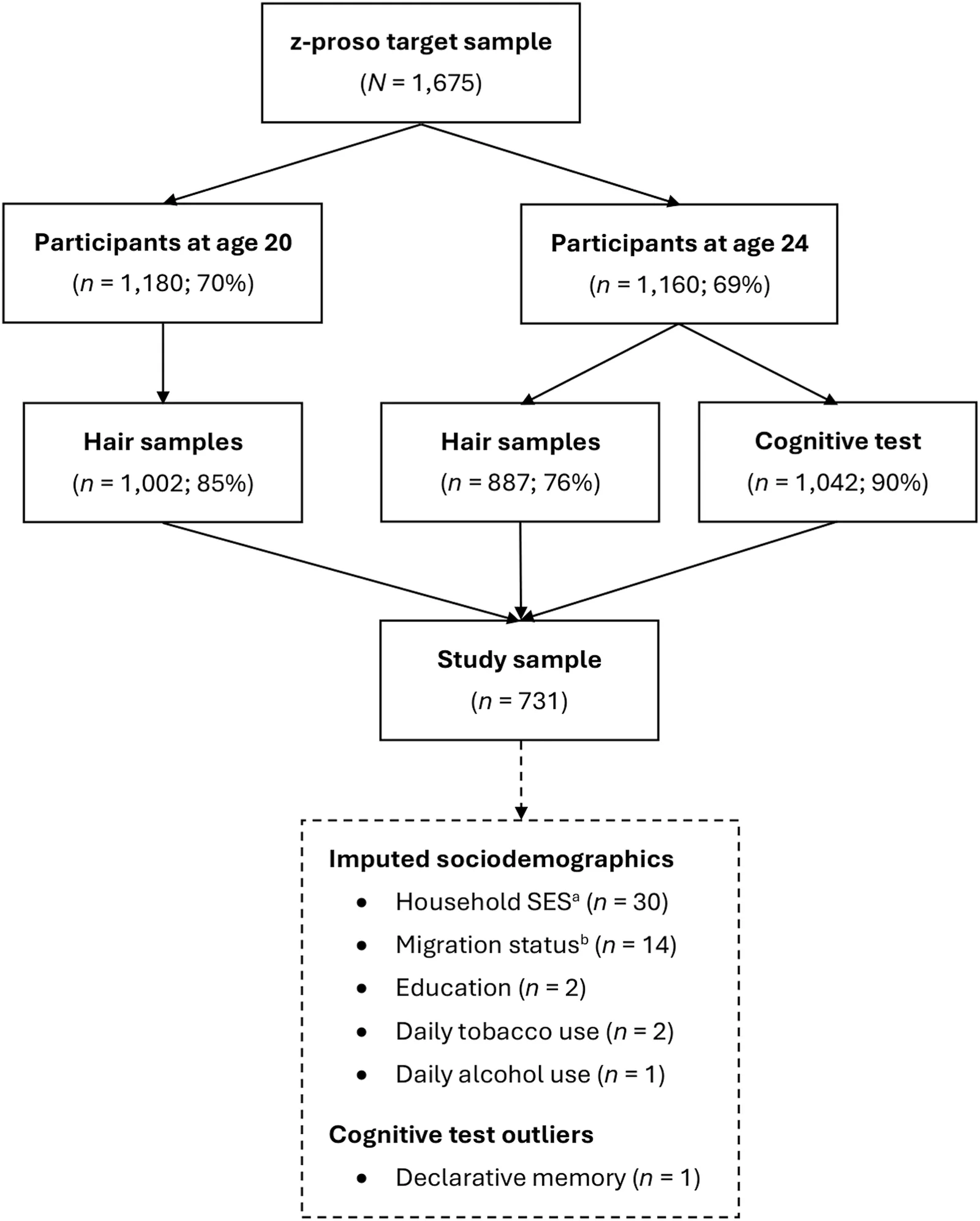
Flow diagram for study sample (*n* = 731). ^*a*^Socioeconomic status (SES) was assessed with the International Socio-Economic Index of Occupational Status, ranging from 14 (unskilled worker) to 90 (judge). ^*b*^Migration background was coded 1 if both parents were not born in Switzerland. z-proso, Zurich Project on Social Development From Childhood to Adulthood.

Among the 1065 participants who took part in both assessment waves, 731 had complete data on hair samples and mental health symptoms across both waves and completed the cognitive tests at age 24 (Figure 1). About half of these participants were female (49.0%), most had completed a secondary education at age 24, and slightly less than half had a migration background (43.9%). Detailed descriptives for the overall analytic sample and each change profile are shown in Table 1. Sample characteristics for the analytic, study, and target samples are presented in Table S5.

**Table 1:** Sample Characteristics Stratified by Substance Use Change Profiles

	Overall, *n* = 731	Nonillicit, *n* = 291	Low/Decreasers, *n* = 288	Cannabis Increasers, *n* = 48	Club-Drug Increasers, *n* = 40	Medical Stimulant Increasers, *n* = 43	Medical Sedative Increasers, *n* = 21
Female Sex	358 (49.0%)	157 (54.0%)	145 (50.3%)	10 (20.8%)	18 (45.0%)	20 (46.5%)	8 (38.1%)
Household SES^a^	47.24 (19.61)	49.02 (18.91)	45.05 (19.63)	51.67 (20.52)	43.50 (20.75)	53.14 (20.21)	37.57 (16.43)
Migration Status, 1^b^	321 (43.9%)	119 (40.9%)	140 (48.6%)	17 (35.4%)	17 (42.5%)	17 (39.5%)	11 (52.4%)
Completed Education							
Below apprenticeship	67 (9.2%)	16 (5.5%)	26 (9.0%)	8 (16.7%)	8 (20.0%)	6 (14.0%)	3 (14.3%)
Apprenticeship	272 (37.2%)	93 (32.0%)	118 (41.0%)	18 (37.5%)	15 (37.5%)	13 (30.2%)	15 (71.4%)
Vocational	182 (24.9%)	78 (26.8%)	75 (26.0%)	10 (20.8%)	9 (22.5%)	9 (20.9%)	1 (4.8%)
Academic	210 (28.7%)	104 (35.7%)	69 (24.0%)	12 (25.0%)	8 (20.0%)	15 (34.9%)	2 (9.5%)
Gaming Experience^c^	2.32 (1.67)	2.11 (1.57)	2.30 (1.65)	2.96 (1.69)	2.58 (1.66)	2.86 (2.01)	2.43 (2.05)
Daily Tobacco Use	240 (32.8%)	63 (21.6%)	103 (35.8%)	24 (50.0%)	21 (52.5%)	19 (44.2%)	10 (47.6%)
Daily Alcohol Use	37 (5.1%)	13 (4.5%)	14 (4.9%)	5 (10.4%)	3 (7.5%)	1 (2.3%)	1 (4.8%)
SBQ Symptoms							
Age 20: anxiety-depressive	2.43 (0.84)	2.32 (0.80)	2.51 (0.90)	2.57 (0.79)	2.52 (0.78)	2.43 (0.65)	2.37 (0.82)
Age 24: anxiety-depressive	2.52 (0.83)	2.38 (0.76)	2.58 (0.87)	2.85 (0.87)	2.62 (0.85)	2.63 (0.72)	2.67 (0.97)
Age 20: ADHD	2.72 (0.76)	2.62 (0.74)	2.78 (0.79)	2.83 (0.62)	2.78 (0.79)	2.90 (0.72)	2.64 (0.71)
Age 24: ADHD	2.86 (0.78)	2.72 (0.75)	2.84 (0.76)	3.10 (0.82)	2.99 (0.78)	3.42 (0.84)	2.89 (0.82)
Age 20: psychotic	1.47 (0.52)	1.41 (0.50)	1.49 (0.51)	1.71 (0.64)	1.56 (0.57)	1.34 (0.37)	1.60 (0.51)
Age 24: psychotic	1.41 (0.50)	1.35 (0.43)	1.40 (0.47)	1.68 (0.71)	1.54 (0.61)	1.40 (0.40)	1.70 (0.90)
CANTAB Score							
Sustained attention, sensitivity	0.91 (0.05)	0.92 (0.05)	0.90 (0.05)	0.90 (0.05)	0.88 (0.06)	0.91 (0.06)	0.86 (0.07)
Working memory, errors	7.13 (7.74)	6.45 (7.06)	7.44 (7.99)	5.73 (7.25)	9.60 (8.66)	6.42 (8.37)	12.19 (8.87)
Declarative memory, errors	6.31 (7.76)	5.68 (6.79)	6.39 (7.79)	6.27 (7.80)	6.60 (7.75)	8.42 (11.54)	9.00 (9.78)

Values are presented as mean (SD) or *n* (%).

CANTAB, Cambridge Neuropsychological Test Automated Battery; SBQ, Social Behavior Questionnaire; SES, socioeconomic status.

a SES assessed with the International Socio-Economic Index of Occupational Status, ranging from 14 (unskilled worker) to 90 (judge).

b Migration background coded as 0 = at least one parent born in Switzerland (negative) and 1 = both parents were not born in Switzerland (positive).

c Frequency of playing action-packed video games in the past year, averaged across ages 20 and 24, coded as (1 = never to 7 = daily).

### Substance Use and Change Profiles

Of the 731 participants, 440 (60.2%) tested positive for at least 1 substance at age 20 or age 24; 107 (14.6%) and 167 (22.9%) tested positive for more than 1 substance (excluding cannabidiol) at ages 20 or 24, respectively (Figure S2). Notably, cocaine use was highly prevalent, with 23.0% at age 24 as reported previously (22). From age 20 to 24, the prevalence rates of most substances increased (Figure S2), with ADHD medication (methylphenidate) increasing the most (+220%). Codeine, an opioid commonly sold as cough syrups, decreased the most (−34%), perhaps due to changes in regulations or age-related drug use preferences.

**Figure 2:**
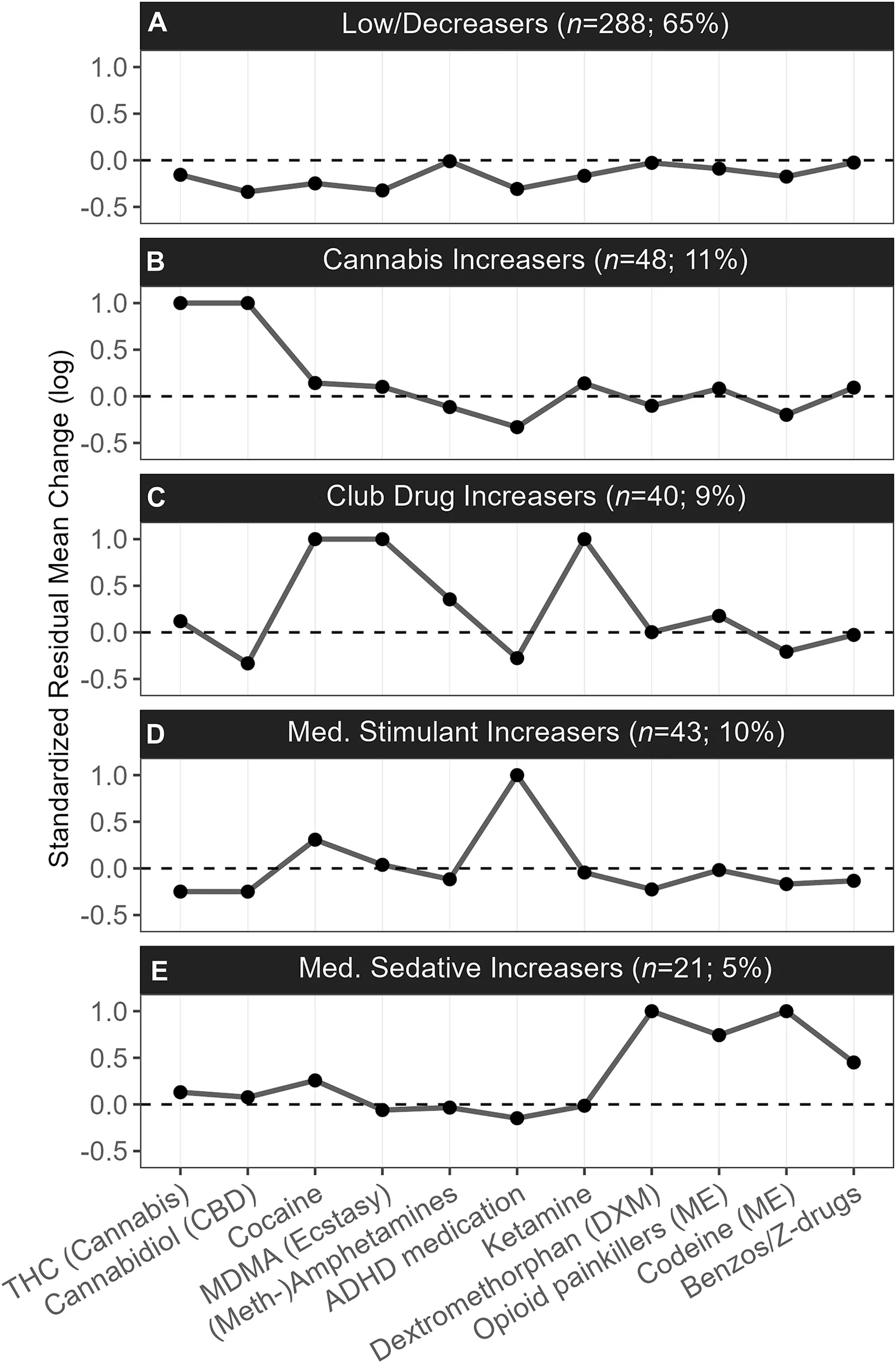
**(A–E)** Changes in substance concentrations between ages 20 and 24, stratified by latent profiles (*n* = 731). Values larger than 1 were set to 1 to facilitate readability. ADHD, attention-deficit/hyperactivity disorder; MDMA, 3,4-methylenedioxymethamphetamine; ME, morphine equivalent; med., medical; THC, Δ^9^-tetrahydrocannabinol.

Among participants with substance use, LPA revealed 5 distinct change profiles (fit indices in Table 2 and profiles in Figure 2), which were labeled as low/decreasers (*n* = 288, 65.5%), cannabis increasers (*n* = 48, 10.9%), club drug increasers (*n* = 40, 9.1%), medical stimulant increasers (*n* = 43, 9.8%), and medical sedative increasers (*n* = 21, 4.8%).

**Table 2:** Fit Indices for Number of Latent Profiles

Number of Profiles	AIC	CAIC	BIC	SABIC	BLRT	*p* Value	Entropy
1	15,641.2	15,753.1	15,731.1	15,661.3	–	–	1.000
2	15,257.6	15,430.6	15,396.6	15,288.7	407.6	<.010	0.971
3	14,923.9	15,157.9	15,111.9	14,965.9	357.8	<.010	0.976
4	14,496.7	14,791.7	14,733.7	14,549.7	451.2	<.010	0.978
5^a^	14,226.6	14,582.6	14,512.6	14,290.5	294.1	<.010	0.976
6	14,308.8	14,725.9	14,643.9	14,383.7	−58.3	.999	0.739

BLRT, bootstrapped likelihood ratio test; CAIC, consistent Akaike information criterion; SABIC, sample size–adjusted Bayesian information criterion.

a Five-profile solution that was found to be the best fit for the data.

### Cognitive Performance Levels

**Figure 3:**
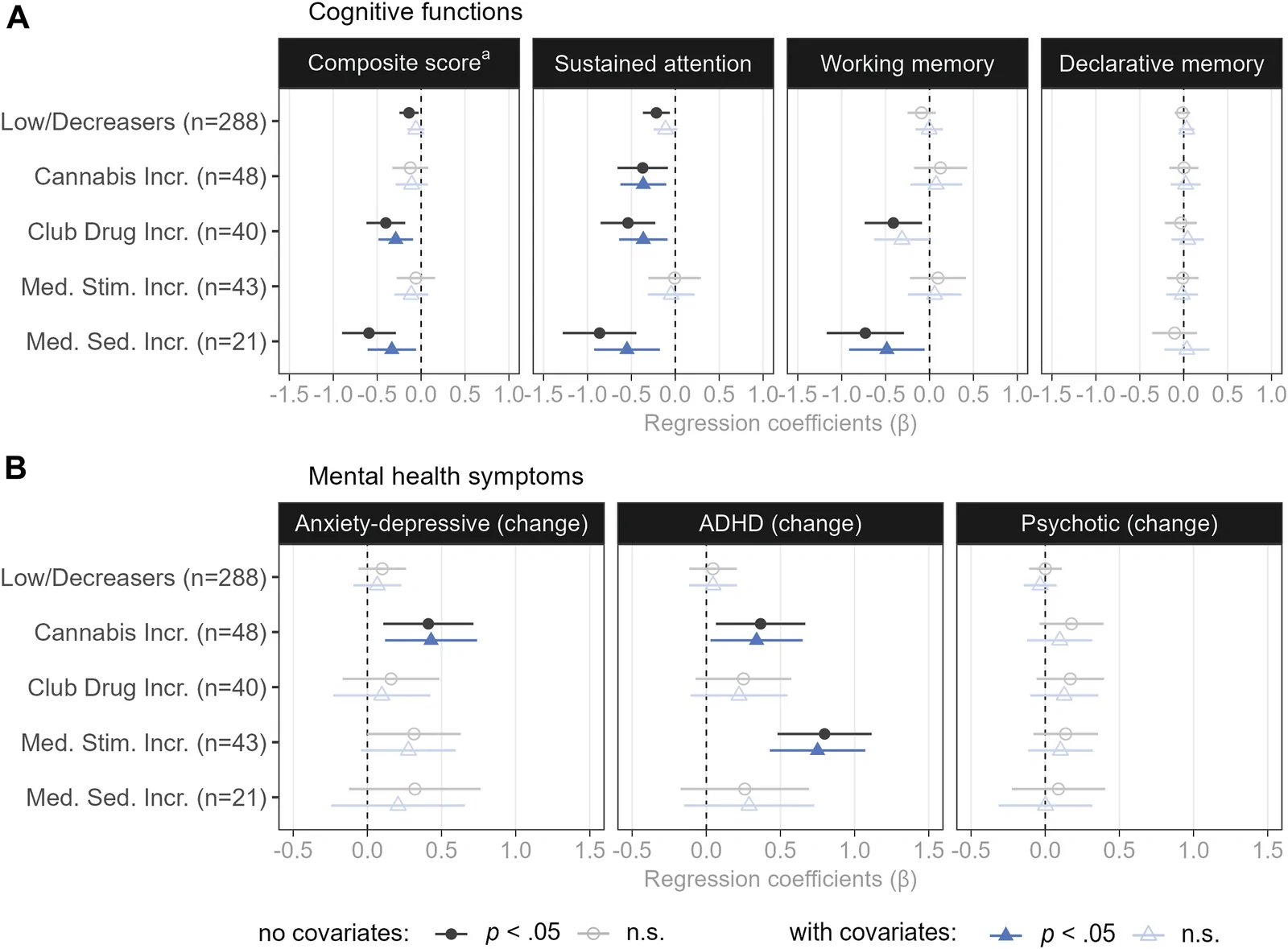
Associations of substance use profiles with **(A)** cognitive functions and **(B)** mental health symptoms in relation to participants with no illicit use (*n* = 291). ^*a*^Composite score reflects mean score of the sustained attention, working memory, and declarative memory task. ADHD, attention-deficit/hyperactivity disorder; incr., increaser; med. sed., medical sedative; med. stim., medical stimulant; n.s., nonsignificant.

Regression analyses were used to examine associations between profiles of change in recent substance use and cognitive performance levels at age 24 (Figure 3A). Compared with participants with no illicit use (*n* = 291, 39.8%) and after controlling for covariates, cannabis increasers showed lower performance on the attention task (β = −0.36; 95% CI, −0.62 to −0.10; *d* = 0.34), and club drug increasers showed lower attention (β = −0.36; 95% CI, −0.64 to −0.09; *d* = 0.41) and a lower composite cognitive score (β = −0.29; 95% CI, −0.49 to −0.09; *d* = 0.39). Medical sedative increasers showed lower attention (β = −0.55, 95% CI, −0.92 to −0.18; *d* = 0.74), working memory (β = −0.48; 95% CI, −0.91 to −0.05; *d* = 0.58), and composite cognitive score performance (β = −0.33; 95% CI, −0.61 to −0.06; *d* = 0.76). None of the profiles of change in recent substance use were significantly associated with declarative memory. No differences in cognitive performance levels were found between the low/decreasers and participants with no illicit use. Detailed model summaries are provided in Table S6.

Because almost half of the medical stimulant increasers who self-reported prescription use of methylphenidate due to behavioral or psychological problems between ages 20 and 24 (*n* = 12 [27.9%] initiated use between ages 20 and 24, *n* = 5 [11.6%] reported use at both ages, and none discontinued use during this period), we conducted post hoc sensitivity analyses including prescribed methylphenidate (yes/no) as an additional confounder. Methylphenidate prescription between ages 20 and 24 showed a positive bivariate association with working memory performance (β = 0.34; 95% CI, 0.04 to 0.64; *d* = 0.37), but no associations were observed for the composite cognitive score, attention, or declarative memory. Adjusting for this confounder in the multivariable models did not affect the associations between the medical stimulant increaser profile and cognitive outcomes; all remained nonsignificant.

### Mental Health Symptom Changes

Regression models assessed changes in mental health symptoms (anxiety-depressive, ADHD, and psychotic-like) in relation to profiles of change in recent substance use between ages 20 and 24 (Figure 3B). Compared with participants with no illicit use, cannabis increasers exhibited a stronger increase in anxiety-depressive symptoms (β = 0.43; 95% CI, 0.12 to 0.74; *d* = 0.53) and a greater increase in ADHD symptoms (β = 0.34; 95% CI, 0.03 to 0.65; *d* = 0.38), with associations persisting after covariate adjustment. Medical stimulant increasers showed a strong increase in ADHD symptoms compared with participants with no illicit use (β = 0.75; 95% CI, 0.43 to 1.07; *d* = 0.82), with associations remaining significant after adjusting for covariates. No significant associations were found for psychotic-like symptoms across substance use profiles. No differences in mental health symptom changes were found between the low/decreasers and participants with no illicit use. Detailed model summaries are provided in Table S7.

Post hoc sensitivity analyses showed that methylphenidate prescription between ages 20 and 24 was bivariately associated with greater rises in ADHD symptoms between ages 20 and 24 (β = 0.35; 95% CI, 0.04 to 0.66; *d* = 0.38). Adjusting for this confounder did not affect the association between the medical stimulant increaser profile and increased ADHD symptoms (β = 0.73; 95% CI, 0.39 to 1.07; *d* = 0.80), and the associations with anxiety-depressive and psychotic-like symptoms remained nonsignificant.

## Discussion

In this longitudinal age cohort of 731 young adults, 60.2% tested positive in hair for at least 1 psychoactive substance at age 20 or 24, with the prevalence of most substances increasing over time. Young adults in profiles with increasing cannabis, club drug (cocaine, MDMA, amphetamines, ketamine), or medical sedative (benzodiazepines, opioid painkillers, DXM) use showed lower cognitive performance, especially in attention and working memory. Increasing cannabis use was also linked to rising anxiety-depressive symptoms, and increasing stimulant use was linked to increasing anxiety-depressive and ADHD symptoms. Importantly, most substance use patterns observed in this sample were likely recreational and did not meet the criteria for substance use disorders; for example, at age 24, 95.8% of participants with cocaine use were below the hair concentration threshold suggested for cocaine dependence (78).

### Substance Use Profiles

The overall increase of substance use is consistent with developmental theories that characterize emerging adulthood as a period of ongoing neurocognitive (4,5) and social (6,79) change, during which experimentation and shifts in use patterns may persist before stabilizing or declining. Importantly, the high and increasing prevalence of testing positive for more than 1 substance in hair from ages 20 to 24 (14.6%–22.9%, respectively) and distinct change profiles indicate substantial heterogeneity in emerging adults’ recent substance use, highlighting the importance of using analytic approaches that capture naturalistic patterns of use rather than focusing on single substances in isolation. The marked increase in medical stimulant use (+220%) may reflect more frequent prescription of these drugs for attentional difficulties among college-age populations, increased nonmedical use for cognitive performance enhancement, or a combination of both (80,81). Finally, the observed increase in the use of club drugs and sedatives is clinically highly relevant as the respective profiles included substances of high addictive potential such as cocaine, opioids, and benzodiazepines (82,83).

### Cognitive Performance Levels, Cannabis, Club Drugs, and Sedatives

Profiles characterized by increasing cannabis, club drug, and sedative use were associated with lower sustained attention and working memory performance at age 24, comparable with previous cross-sectional findings in a similar population. Furthermore, this pattern is consistent with experimental and neuroimaging evidence indicating that THC, cocaine, MDMA, ketamine, and opioid analgesics affect cortical systems central to these cognitive domains (26,28,29,34,84,85). Given that specifically prefrontal systems supporting attention and working memory continue to mature well into the mid-20s (3,86), substance exposure during this developmental period may increase the likelihood of measurable cognitive differences, even at predominantly recreational levels of use. Notably, visuospatial declarative memory appeared largely unaffected, suggesting either greater resilience of this function or a lower sensitivity of the task to recreational exposure levels. Verbal declarative memory tasks have shown greater sensitivity to the effects of substance use (87), especially regarding cannabis (88).

From a clinical perspective, even modest cognitive differences can carry functional consequences during emerging adulthood, including reduced academic and school performance, diminished productivity, and greater difficulty managing everyday cognitive demands (89,90). They are also associated with increased risks in driving and other potentially dangerous activities (91,92) and could exacerbate psychiatric comorbidities or reinforce maladaptive substance use trajectories (93,94). It is important to acknowledge that cognitive differences observed at age 24 may reflect premorbid characteristics rather than consequences of substance exposure, as lower attentional and working memory capacity could precede and contribute to substance use initiation and escalation through impaired inhibitory control or shared neurobiological vulnerabilities (95,96). Establishing a clear temporal ordering will require longitudinal designs that continuously track substance exposure, cognition, and contextual factors from before the onset of use to disentangle premorbid cognitive profiles from exposure-related change.

### Mental Health Symptom Changes, Cannabis, and Medical Stimulants

Increasing cannabis use was associated with rising anxiety-depressive and ADHD symptoms, consistent with prior research documenting mixed but non-negligible associations between cannabis use and mental health symptoms (39, 40, 41,97). Cannabis modulates neural systems involved in mood and attention regulation (98). Evidence of mood improvements following reduced cannabis use in major depressive disorder (99) together with prospective links between adolescent cannabis use and adult ADHD symptoms (100,101) support the plausibility of direct effects on symptom change. At the same time, young adults with emotional or attentional difficulties are more likely to increase cannabis use as a coping strategy (102,103). Self-medication of ADHD symptoms with cannabis is also common (104), despite meta-analytic evidence indicating that cannabis does not improve ADHD symptoms (105). Taken together, our findings are most consistent with a dual-pathway model in which cannabis-related effects on psychopathology and vulnerability-related increases in use operate simultaneously, consistent with robust evidence for bidirectional associations (106, 107).

The increase in ADHD symptoms among medical stimulant increasers can be interpreted in a similar bidirectional fashion. Higher academic demands encountered in early adulthood may exacerbate attentional difficulties, potentially prompting increased use of prescribed stimulants (108,109). At the same time, nonmedical use of prescription stimulants may worsen inattentive and impulsive behaviors and is often comorbid with anxiety or other substance use disorders (110). Furthermore, neuroimaging studies suggest that chronic stimulant exposure is associated with greater inattentiveness (111) and impulsivity (112), as well as structural and functional changes in brain regions associated with these functions (84). In the current study, the association between increasing medical stimulant use and increasing ADHD symptoms remained largely unchanged after adjusting for self-reported prescription of ADHD medication, suggesting that this association cannot be fully attributed to treatment seeking or the prescription stimulant medication for worsening ADHD symptoms. Instead, they are consistent with the possibility that nonmedical stimulant use may contribute to increases in ADHD symptoms. Although medical stimulant increasers did not show differences in attentional performance at age 24, the only available assessment does not allow conclusions about changes in cognitive functioning over time.

Overall, our findings highlight the need for longitudinal within-person research using clinically validated symptom measures to disentangle temporal ordering and identify whether increasing cannabis and medical stimulant use, worsening symptoms, or shared underlying vulnerabilities primarily drive their co-occurrence.

### Strengths and Limitations

Strengths of this study include the use of objective hair testing to assess cumulative substance exposure, a community-based cohort, and a multivariate approach that captures naturalistic polysubstance patterns. Limitations include the absence of baseline cognitive assessments; a purely behavioral, nonclinical assessment of mental health symptoms; and the lack of detailed information on motives, frequency, and concurrent polysubstance use. Although hair toxicology provides cumulative exposure, exact timing relative to symptom changes is not possible. Because hair toxicology only captures recent exposure windows prior to each assessment, our LPA models cannot account for participants’ substance use histories before age 20 or behavioral fluctuations occurring outside the two 3-month detection windows. Additionally, alcohol and nicotine were not included as LPA indicators because ethyl glucuronide and cotinine were not measured in hair. Future studies would benefit from incorporating these biological markers to provide a more exhaustive analysis of emerging adult substance use. Lastly, latent profile solutions are sample dependent and require replication, and their generalizability to clinical populations or to individuals with higher severity of use is limited. Although the 5-profile solution is strongly supported by model fit and conceptually meaningful, the small profile size of the medical sedative increasers (*n* = 21, 4.8% of the total LPA sample) limits the generalizability of our findings.

### Conclusions

We identified 5 distinct profiles of change in substance use during emerging adulthood. Notably, even profiles characterized by increasing recreational use were associated with lower cognitive performance levels and worsening mental health symptoms. These findings suggest that objective monitoring of substance exposure may help identify individuals at risk for adverse developmental outcomes. Such approaches could also inform targeted prevention and intervention strategies that address both self-regulatory difficulties and escalating substance use during this sensitive developmental period.

## Supplementary Material

Figures S1–S3 and Tables S1–S7

Key Resources Table
