# Supplementary material for: Changing Patterns of Recent Substance Use in Emerging Adulthood and Their Associations With Cognitive Functions and Mental Health
Source: Biol Psychiatry Glob Open Sci. 2026 Jul 24;6(6):100795. doi: 10.1016/j.bpsgos.2026.100795 (PMC13571240; doi:10.1016/j.bpsgos.2026.100795)
Supplement: Supplementary file 1 [file mmc1.pdf]

SUPPLEMENTARY INFORMATION

Changing Patterns of Recent Substance Use in Emerging Adulthood and Their Associations With Cognitive Functions and Mental Health

Eggenberger *et al.*

Figure S1
Outlier overview in CANTAB data (n = 1,042)

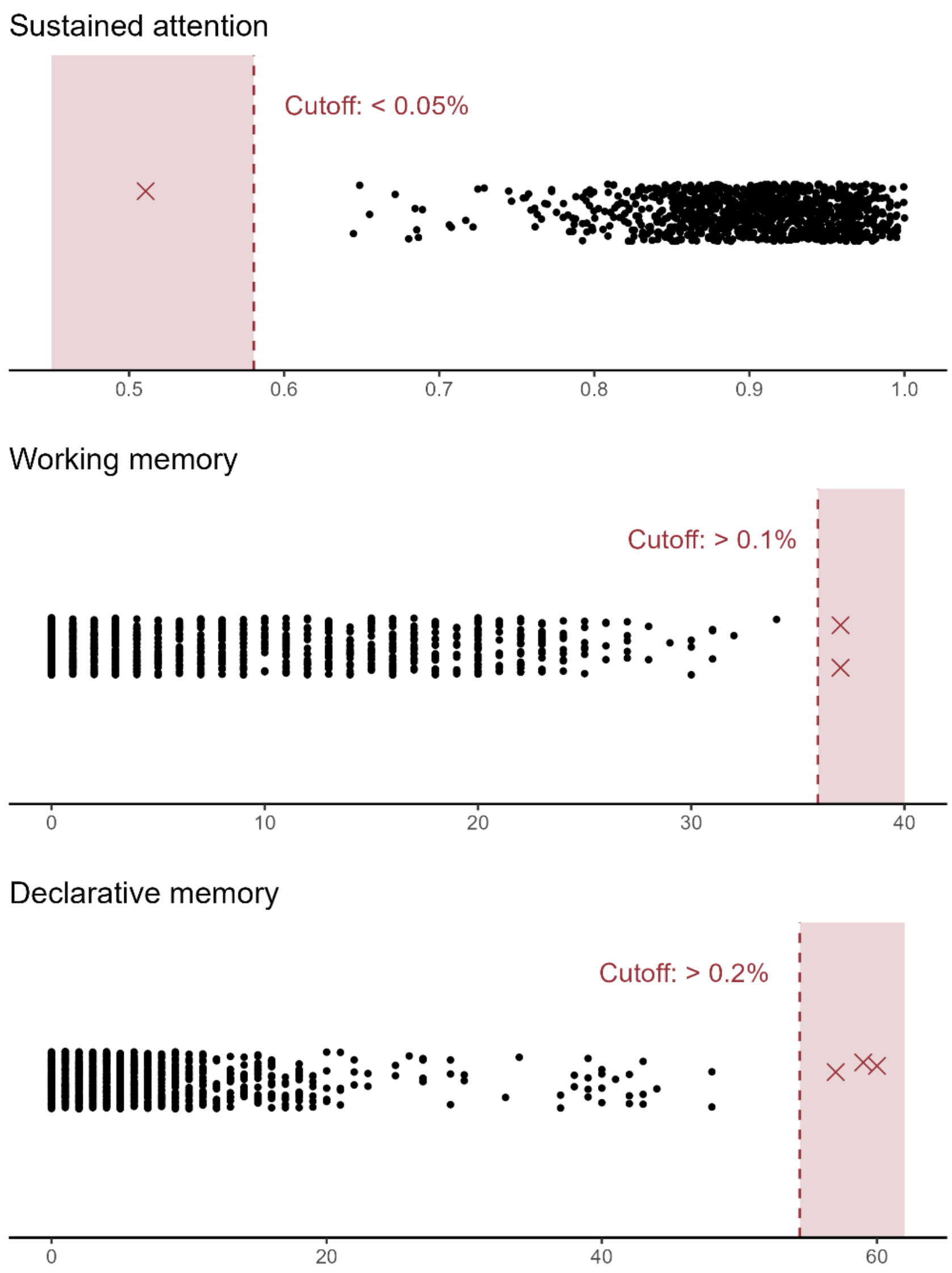

Note. For the cutoff, values below the 0.1st percentile were excluded, applying a tolerance factor of 2 (i.e., data between the 0.05th and 0.2nd percentiles) based on visual inspection of the distributions

Figure S2

Pairwise substance use co-occurrence at ages 20 and 24

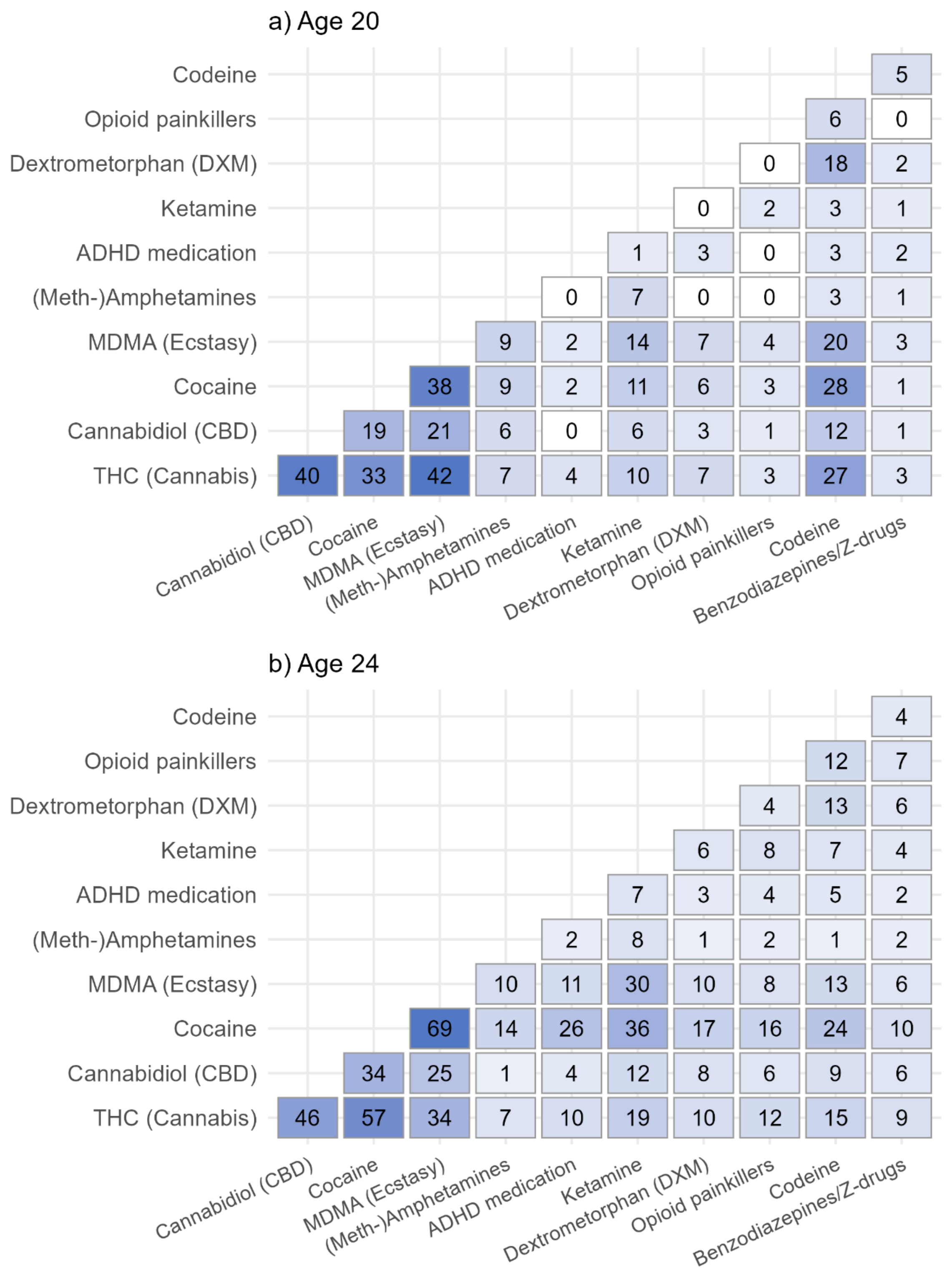

Note. Values indicate number of pairwise co-occurrence.

Figure S3

Substance prevalence rates and relative changes from age 20 to 24 (n=731)

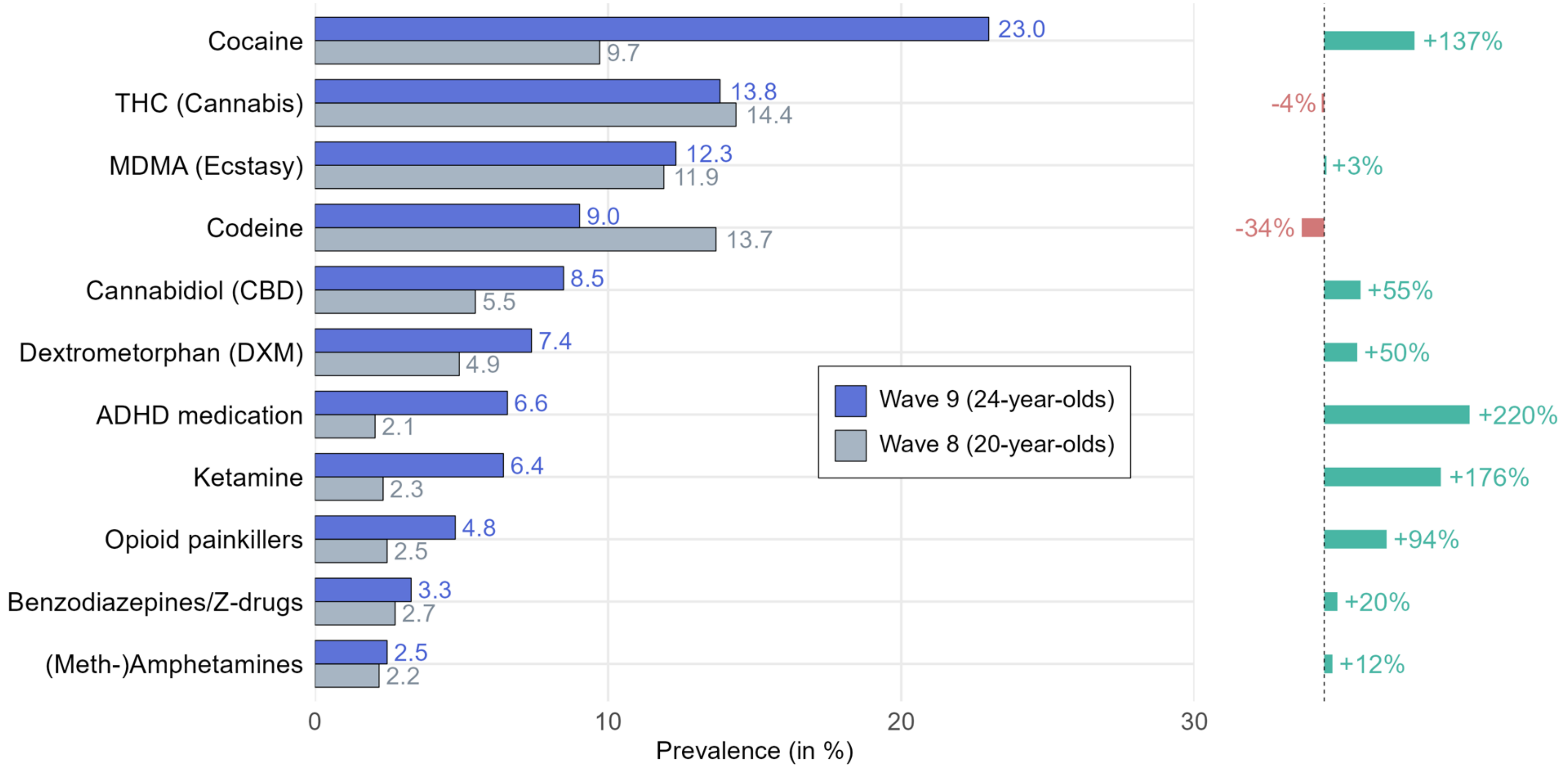

Note. n = number of participants

Table S1
Monetary compensation (in Swiss Francs; CHF) for participation at age 20 and age 24

Module	Age 20	Age 24	
	Lab	*Lab*	*Online*
Survey	75.-	150.-	100.-
Hair sample	30.-	30.-	50.-
Cognitive test	–	50.-	40.-

Table S2
Psychometric properties of the SBQ scales in the study sample (n=731)

	Age 20 (wave 8)			Age 24 (wave 9)			n_{items}	ICC
	M (*SD*)	Median (min; max)	α	*M* (*SD*)	Median (min; max)	α		
**Anxiety-Depressive**	2.43 (0.84)	2.25 (1; 5)	.87	2.52 (0.83)	2.38 (1; 5)	.86	8	.73
**ADHD**	2.72 (0.76)	2.75 (1; 5)	.79	2.86 (0.78)	2.75 (1; 5)	.80	4	.75
**Psychotic**	1.47 (0.52)	1.33 (1; 5)	.68	1.41 (0.50)	1.33 (1; 4.5)	.70	6	.58

Note. *M* = mean, *SD* = standard deviation, α = Cronbach's alpha (reliability), n_{items} = number of items per scale, ICC = intraclass correlation coefficient (stability)

Table S3
Substances, metabolites, and morphine equivalency (ME) factors

Substance category	Substances and metabolites	ME factor
Cannabis (THC)	Tetrahydrocannabinol (THC)<br>Cannabinol (CBN)[a]	
Cannabidiol (CBD)	Cannabidiol (CBD)	
Cocaine	Cocaine<br>Benzoylecgonine[a]<br>Norcocaine[a]<br>Cocaethylene[a]<br>*m*-Hydroxycocaine[a]<br>*p*-Hydroxycocaine[a]	
MDMA („Ecstasy")	3,4-Methylenedioxymethamphetamine (MDMA)<br>3,4-Methylenedioxyamphetamine (MDA)[a]	
ADHD medication	Methylphenidate	
Opioid painkillers [ME]	Morphine	1.000
	Fentanyl	128.333
	Pethidine	0.125
	Tapentadol	0.275
	Tramadol	0.116
	n-Desmethyltramadol[a]	0.116
	Methadone	2.800
	2-Ethylidene-1,5-dimethyl-3,3-diphenylpyrrolidine (EDDP)[a]	2.800
	Oxycodone	1.663
	Noroxycodone[a]	1.663
Codeine [ME]	Codeine	0.145
	Dihydrocodeine	0.231
Ketamine	Ketamine<br>Norketamine[a]	
Dextromethorphan (DXM)	Dextromethorphan (DXM)	

Note. Morphine equivalency (ME) factors were adopted courtesy of Kroll et al. (2018) and Nissen et al. (2001).
[a] metabolite

Table S4
List of covariates and how they were computed from the content of the initial questions (Q), corresponding answer coding (A), assessment wave (W), and informant (I)

Sex assigned-at-birth
- Q: Information on participant's sex
- A: 0 = *male*; 1 = *female*
- W: Wave 1 (age 7)
- I: Primary caregiver

Household SES
- Q: Information on participant's primary caregivers' employment, which was subsequently mapped onto the Socio-Economic Index of Occupational Status (ISEI)
- A: Ranges from 16 = *unskilled worker* to 90 = *judge*
- W: Coalesced across wave 4, 5 and 6 (ages 11, 13, and 15)
- I: Participant

Migration background
- Q: Information on country of birth of participant's biological mother and father
- A: 0 = *at least one parent born in Switzerland*; 1 = *both parents not born in Switzerland* (original answer coding was 1 = *Switzerland*; 2 = *a different country, namely: [free text option]*)
- W: Coalesced across wave 5 and 6 (ages 13 and 15)
- I: Participant

Highest education
- Q: Information on participant's highest completed education
- A: 1 = *below apprenticeship*, 2 = *apprenticeship*, 3 = *vocational tertiary*, 4 = *academic tertiary* (original answer coding ranged from 1 = *compulsory* to 19 = *PhD/Doctorate*)
- W: Wave 9 (age 24)
- I: Participant

Gaming experience
- Q: Information on participant's time spent playing "action-packed computer or video games, in which opponents are realistically killed or blood is depicted ("shooter" games, etc.)" in the last 12 months
- A: Ranges from 1 = *never* to 7 = *daily*
- W: Averaged across wave 8 and 9 (ages 20 and 24)
- I: Participant

Daily tobacco use
- Q: Information on the frequency of participant's tobacco use in the past three months
- A: 0 = *none or less than daily use*; 1 = *daily use* (original answer coding ranged from 1 = *never* to 5 = *daily*)
- W: Wave 9 (age 24)
- I: Participant

Daily alcohol use
- Q: Information on the frequency of participant's alcohol use in the past three months
- A: 0 = *none or less than daily use*; 1 = *daily use* (original answer coding ranged from 1 = *never* to 5 = *daily*)
- W: Wave 9 (age 24)
- I: Participant

Table S5
Sample characteristics stratified by sample type

	**Analytic sample** (*n*=731)	**Study sample** (*n*=1065)	**z-proso target sample** (*n*=1675)
**Female sex**, *n (%)*	358 (49.0)	548 (51.5)	805 (48.1)
**Household SES**[a], *mean (SD)*	47.48 (19.89)	47.93 (19.69)	45.62 (19.30)
**Migration status**[b], *n (%)*	316 (44.1)	474 (45.4)	716 (49.6)
**Completed education**, *n (%)*			
Below apprenticeship	67 (9.2)	90 (8.5)	103 (8.9)
Apprenticeship	270 (37.0)	376 (35.4)	427 (36.9)
Vocational	182 (25.0)	288 (27.1)	313 (27.0)
Academic	210 (28.8)	309 (29.1)	315 (27.2)
**Gaming experience**[c], *mean (SD)*	2.32 (1.67)	2.28 (1.66)	2.35 (1.76)
**Daily tobacco use**, *n (%)*	239 (32.8)	339 (32.0)	377 (32.6)
**Daily alcohol use**, *n (%)*	37 (5.1)	60 (5.6)	61 (5.3)
**SBQ**[d] **symptoms,** *mean (SD)*			
Age 20: Anxiety-depressive	2.43 (0.84)	2.40 (0.82)	2.40 (0.82)
Age 24: Anxiety-depressive	2.52 (0.83)	2.47 (0.81)	2.47 (0.81)
Age 20: ADHD	2.72 (0.76)	2.70 (0.75)	2.69 (0.76)
Age 24: ADHD	2.86 (0.78)	2.81 (0.78)	2.80 (0.78)
Age 20: Psychotic	1.47 (0.52)	1.45 (0.51)	1.47 (0.52)
Age 24: Psychotic	1.41 (0.50)	1.39 (0.49)	1.40 (0.49)

Note.
[a] Socioeconomic status assessed with the International Socio-Economic Index of Occupational Status (ISEI), ranging from 14 (unskilled worker) to 90 (judge).
[b] Migration background coded 1 if both parents were not born in Switzerland.
[c] Frequency of playing action-packed video games in the past year, averaged across ages 20 and 24.
[d] Social Behavior Questionnaire (ADHD = Attention-deficit/hyperacticity disorder).

Table S6
Regression models for substance use profiles and cognitive functions

Predictor	β	95%-CI	*p*-value	Model fit
**A) Outcome: Composite cognitive score**				
Low/Decreasers (n=288)	-0.06	[-0.16; 0.04]	.221	
Cannabis Incr. (n=48)	-0.11	[-0.29; 0.08]	.257	
Club-drug Incr. (n=40)	-0.29	[-0.49; -0.09]	**.004****	
Med. Stim. Incr. (n=43)	-0.11	[-0.30; 0.08]	.254	
Med. Sed. Incr. (n=21)	-0.33	[-0.61; -0.06]	**.018***	
Female sex	-0.04	[-0.14; 0.07]	.512	
Household SES[a]	0.05	[-0.01; 0.10]	.079	
Migration background[b]	-0.17	[-0.27; -0.08]	**<.001*****	
Education: below apprenticeship	-0.26	[-0.42; -0.10]	**.002****	
Education: vocational	0.25	[0.14; 0.37]	**<.001*****	
Education: academic	0.50	[0.38; 0.63]	**<.001*****	
Gaming experience[c]	0.11	[0.06; 0.16]	**<.001*****	
Daily tobacco use	0.03	[-0.07; 0.13]	.512	
Daily alcohol use	0.07	[-0.13; 0.27]	.500	
				R^2 (adj.) = .24, RSE = .59
**B) Outcome: Sustained attention**				
Low/Decreasers (n=288)	-0.11	[-0.25; 0.03]	.110	
Cannabis Incr. (n=48)	-0.36	[-0.62; -0.10]	**.006****	
Club-drug Incr. (n=40)	-0.36	[-0.64; -0.09]	**.010****	
Med. Stim. Incr. (n=43)	-0.05	[-0.31; 0.22]	.729	
Med. Sed. Incr. (n=21)	-0.55	[-0.92; -0.18]	**.004****	
Female sex	-0.12	[-0.27; 0.04]	.133	
Household SES[a]	0.08	[0.00; 0.15]	**.048***	
Migration background[b]	-0.14	[-0.27; 0.00]	**.048***	
Education: below apprenticeship	-0.51	[-0.74; -0.28]	**<.001*****	
Education: vocational	0.30	[0.14; 0.46]	**<.001*****	
Education: academic	0.75	[0.58; 0.92]	**<.001*****	
Gaming experience[c]	0.12	[0.04; 0.19]	**.003****	
Daily tobacco use	0.07	[-0.07; 0.21]	.305	
Daily alcohol use	-0.08	[-0.37; 0.20]	.568	
				R^2 (adj.) = .26, RSE = .81
**C) Outcome: Working memory**				
Low/Decreasers (n=288)	0.00	[-0.16; 0.15]	.958	
Cannabis Incr. (n=48)	0.08	[-0.22; 0.37]	.613	
Club-drug Incr. (n=40)	-0.31	[-0.63; 0.00]	.052	
Med. Stim. Incr. (n=43)	0.06	[-0.25; 0.36]	.707	
Med. Sed. Incr. (n=21)	-0.49	[-0.92; -0.06]	**.027***	
Female sex	-0.13	[-0.30; 0.04]	.128	
Household SES[a]	0.05	[-0.04; 0.13]	.262	
Migration background[b]	-0.25	[-0.40; -0.10]	**.001****	
Education: below apprenticeship	-0.19	[-0.44; 0.06]	.143	
Education: vocational	0.26	[0.08; 0.45]	**.006****	
Education: academic	0.44	[0.24; 0.64]	**<.001*****	
Gaming experience[c]	0.11	[0.02; 0.19]	**.016***	
Daily tobacco use	0.01	[-0.15; 0.17]	.901	

Table S6

Regression models for substance use profiles and cognitive functions

Predictor	β	95%-CI	*p*-value	Model fit
Daily alcohol use	0.25	[-0.07; 0.58]	.129	
				R^2 (adj.) = .12, RSE = 0.92
**D) Outcome: Declarative memory**				
Low/Decreasers (n=288)	0.03	[-0.06; 0.12]	.470	
Cannabis Incr. (n=48)	0.02	[-0.15; 0.19]	.801	
Club-drug Incr. (n=40)	0.05	[-0.14; 0.23]	.628	
Med. Stim. Incr. (n=43)	-0.02	[-0.20; 0.16]	.850	
Med. Sed. Incr. (n=21)	0.04	[-0.22; 0.29]	.783	
Female sex	-0.03	[-0.13; 0.07]	.510	
Household SES[a]	0.03	[-0.01; 0.08]	.165	
Migration background[b]	-0.09	[-0.18; 0.00]	**.043***	
Education: below apprenticeship	-0.04	[-0.19; 0.12]	.640	
Education: vocational	0.13	[0.02; 0.24]	**.017***	
Education: academic	0.27	[0.16; 0.38]	**<.001*****	
Gaming experience[c]	0.04	[-0.01; 0.09]	.083	
Daily tobacco use	-0.02	[-0.11; 0.07]	.675	
Daily alcohol use	0.05	[-0.14; 0.23]	.621	
				R^2 (adj.) = .06, RSE = .55

Note. Reference category for education is below apprenticeship. Decr. = decreaser, incr. = increaser, med. stim. = medical stimulant, med. sed. = medical sedative, CI = confidence interval, RSE = residual standard error

[a] Socioeconomic status assessed with the International Socio-Economic Index of Occupational Status (ISEI), ranging from 14 (unskilled worker) to 90 (judge).

[b] Positive migration background if both parents were not born in Switzerland.

[c] Frequency of playing action-packed video games in the past year, averaged across ages 20 and 24.

Table S7

Regression models for substance use profiles and mental health symptoms

Predictor	β	95%-CI	*p*-value	Model fit
**A) Outcome: Anxiety-depressive (change)**				
Low/Decreasers (n=288)	0.07	[-0.09; 0.23]	.415	
Cannabis Incr. (n=48)	0.43	[0.12; 0.74]	**.007****	
Club-drug Incr. (n=40)	0.10	[-0.23; 0.42]	.561	
Med. Stim. Incr. (n=43)	0.28	[-0.04; 0.60]	.089	
Med. Sed. Incr. (n=21)	0.21	[-0.24; 0.66]	.369	
Female sex	0.28	[0.10; 0.46]	**.002****	
Household SES[a]	-0.03	[-0.12; 0.06]	.538	
Migration background[b]	0.10	[-0.06; 0.26]	.201	
Education: below apprenticeship	0.12	[-0.15; 0.39]	.389	
Education: vocational	0.07	[-0.12; 0.26]	.483	
Education: academic	0.04	[-0.16; 0.25]	.676	
Gaming experience[c]	0.03	[-0.06; 0.12]	.516	
Daily tobacco use	0.26	[0.09; 0.42]	**.002****	
Daily alcohol use	-0.07	[-0.41; 0.27]	.683	
				R^2 (adj.) = .04, RSE = .96
**B) Outcome: ADHD (change)**				
Low/Decreasers (n=288)	0.05	[-0.12; 0.21]	.583	
Cannabis Incr. (n=48)	0.34	[0.03; 0.65]	**.032***	
Club-drug Incr. (n=40)	0.22	[-0.11; 0.55]	.185	
Med. Stim. Incr. (n=43)	0.75	[0.43; 1.07]	**<.001*****	
Med. Sed. Incr. (n=21)	0.29	[-0.15; 0.73]	.197	
Female sex	0.13	[-0.05; 0.31]	.161	
Household SES[a]	0.03	[-0.06; 0.12]	.500	
Migration background[b]	-0.03	[-0.19; 0.13]	.750	
Education: below apprenticeship	0.34	[0.07; 0.60]	**.014***	
Education: vocational	0.01	[-0.19; 0.20]	.940	
Education: academic	0.15	[-0.06; 0.35]	.158	
Gaming experience[c]	0.04	[-0.05; 0.13]	.405	
Daily tobacco use	0.06	[-0.11; 0.22]	.500	
Daily alcohol use	0.21	[-0.13; 0.55]	.228	
				R^2 (adj.) = .04, RSE = .96
**C) Outcome: Psychotic (change)**				
Low/Decreasers (n=288)	-0.03	[-0.14; 0.08]	.545	
Cannabis Incr. (n=48)	0.10	[-0.12; 0.32]	.383	
Club-drug Incr. (n=40)	0.13	[-0.10; 0.36]	.274	
Med. Stim. Incr. (n=43)	0.10	[-0.12; 0.32]	.357	
Med. Sed. Incr. (n=21)	0.00	[-0.31; 0.32]	.990	
Female sex	-0.10	[-0.22; 0.02]	.114	
Household SES[a]	0.04	[-0.02; 0.11]	.148	
Migration background[b]	0.13	[0.02; 0.24]	**.017***	
Education: below apprenticeship	-0.02	[-0.21; 0.17]	.829	
Education: vocational	-0.17	[-0.30; -0.03]	**.015***	
Education: academic	-0.13	[-0.27; 0.01]	.068	
Gaming experience[c]	0.02	[-0.05; 0.08]	.614	
Daily tobacco use	0.03	[-0.08; 0.15]	.595	
Daily alcohol use	0.07	[-0.17; 0.30]	.589	
				R^2 (adj.) = .01, RSE = .68

Note. Reference category for education is below apprenticeship. Decr. = decreaser, incr. = increaser, med. stim. = medical stimulant, med. sed. = medical sedative, CI = confidence interval, RSE = residual standard error

[a] Socioeconomic status assessed with the International Socio-Economic Index of Occupational

Status (ISEI), ranging from 14 (unskilled worker) to 90 (judge).
[b] Positive migration background if both parents were not born in Switzerland.
[c] Frequency of playing action-packed video games in the past year, averaged across ages 20 and 24.
